# Multicomponent Strategies to Prevent SARS-CoV-2 Transmission — Nine Overnight Youth Summer Camps, United States, June–August 2021

**DOI:** 10.15585/mmwr.mm7040e1

**Published:** 2021-10-08

**Authors:** Kim Van Naarden Braun, Mark Drexler, Ranna A. Rozenfeld, Eytan Deener-Agus, Rebecca Greenstein, Michael Agus, Mark Joffe, Andrea Kasowitz, Philip Levy, Cliff Nerwen

**Affiliations:** ^1^Ramah Camping Movement, New York City, New York; ^2^National Ramah Medical Committee; ^3^NorthShore University Health Systems, Evanston, Illinois; ^4^Hasbro Children’s Hospital, The Warren Alpert Medical School of Brown University, Providence, Rhode Island; ^5^Boston Children’s Hospital, Harvard Medical School, Boston, Massachusetts; ^6^Children’s Hospital of Philadelphia, University of Pennsylvania, Philadelphia; ^7^Cohen Children’s Medical Center, Zucker School of Medicine at Hofstra/Northwell, Hempstead, New York.

Most U.S. overnight youth camps did not operate during the summer of 2020 because of the COVID-19 pandemic* (*1*). Several that did operate demonstrated that multiple prevention strategies, including pre- and postarrival testing for SARS-CoV-2, the virus that causes COVID-19, masking, and physical distancing helped prevent the introduction and spread of COVID-19; in contrast, camps that relaxed prevention strategies, such as requiring a single prearrival test without subsequent testing, experienced outbreaks (*2*–*4*). The availability of COVID-19 vaccines for persons aged ≥12 years enabled implementation of an additional prevention strategy that was not available in summer 2020. This study assessed the number of COVID-19 cases and potential secondary spread among 7,173 staff members and campers from 50 states, 13 countries, and U.S. military overseas bases at nine independently operated U.S. summer youth camps affiliated with the same organization. The camps implemented multiple prevention strategies including vaccination, testing, podding (cohorting), masking, physical distancing, and hand hygiene during June–August 2021. Vaccination coverage was 93% among eligible persons aged ≥12 years.^†^ All staff members (1,955) and campers (5,218) received site-specific, protocol-defined screening testing, which included prearrival testing and screening tests during the camp session (38,059 tests). Screening testing identified six confirmed COVID-19 cases (one in a staff member and five in campers) by reverse transcription–polymerase chain reaction (RT-PCR) testing (screening test positivity rate = 0.02%). Three additional cases (in two staff members and one camper) were identified based on symptoms and were confirmed by RT-PCR testing. Testing for SARS-CoV-2, isolation, and quarantine in a population with high vaccination coverage resulted in no known secondary transmission of SARS-CoV-2 identified during camp. Implementation of multicomponent strategies is critical for prevention of COVID-19 outbreaks in congregate settings, including overnight youth camps.

During 2021, each of the nine affiliated camps designed site-specific COVID-19 protocols with guidance from their organization’s national medical committee, CDC, the American Academy of Pediatrics, the American Camp Association, and state and local health departments (*5*–*7*). In March 2021, camp staff members became eligible for vaccination as group 1b (frontline essential workers) (*8*). All camps strongly recommended vaccination for eligible persons; seven of nine camps required staff members aged ≥17 years to be fully vaccinated before camp arrival. Data collection for this study included documentation of COVID-19 protocols, demographic and vaccination characteristics of camp populations, results of SARS-CoV-2 screening testing, characteristics of persons who received positive test results, and actions taken in response to cases. Deidentified demographic information, testing counts, and descriptions of positive tests and confirmed cases were submitted via a secure portal. Institutional Review Board approval and waiver of informed consent were granted through NorthShore University HealthSystems (Evanston, Illinois).

Physical camp locations were in the New England (two), Middle Atlantic (two), South (one), Midwest (one), and West (three) U.S. Census regions/divisions. Camp session duration (range = 2–8 weeks [eight camps had multiple sessions]) and size (300–1,130 persons) varied (Table 1). Seven camps had an intersession (1–14 days) between two or more sessions. All 7,173 persons attending the nine camps during June–August 2021 were included in the study, including 5,218 (73%) and 1,955 (27%) staff members. Approximately 30% of persons were aged <12 years and thus ineligible for COVID-19 vaccination. Among eight camps with vaccination data, 4,000 (65%) of all 6,135 persons were vaccinated, including 93% of age-eligible persons (aged ≥12 years), 88% of persons aged 12–16 years, and 99% of those aged ≥17 years.

**TABLE 1:** Demographic characteristics and vaccination status of campers and staff members, camp characteristics, and local SARS-CoV-2 community transmission — nine U.S. overnight camps, June–August 2021

Characteristic	Camp (total no. of campers and staff members), %									
	Total (N = 7,173)	A (n = 677)	B (n = 1,062)	C (n = 1,130)	D (n = 300)	E (n = 831)	F (n = 1,038)	G (n = 525)	H (n = 490)	I (n = 1,120)
**Camp population**										
Campers	**72.8**	68.1	76.7	72.5	77.0	62.7	71.9	69.9	80.0	77.3
Staff members	**27.2**	31.9	23.3	27.5	23.0	37.3	28.1	30.1	20.0	22.7
**Sex**										
Male	**46.4**	43.6	45.5	44.8	69.7	47.2	46.3	50.1	42.2	43.8
Female	**53.5**	55.8	54.5	54.7	30.3	52.8	53.7	49.7	57.8	56.3
Undefined	**0.1**	0.6	0.0	0.5	0.0	0.0	0.0	0.2	0.0	0.0
Age group, yrs* (all campers/staff members)										
<12	**30.1**	24.2	27.0	37.2	22.3	35.1	30.2	27.6	28.6	29.4
12–16	**37.8**	42.0	46.7	31.8	52.3	23.2	37.1	34.3	49.4	37.1
≥17	**32.1**	33.8	26.3	31.0	25.3	41.7	32.7	38.1	22.0	33.5
**Age group, yrs (vaccinated^†^ campers/staff members [n = 6,135])**										
<12	**0.1**	0.0	0.0	0.0	1.5	0.0	NR	0.7	0.0	0.0
12–16	**88.1**	91.5	94.6	85.0	89.2	88.6	NR	77.8	81.0	88.5
≥17	**99.3**	99.1	100.0	99.7	100.0	99.4	NR	97.5	99.1	99.2
**Region/Division**^§^ **of camper or staff member home residence**										
New England	**8.0**	0.4	42.2	1.9	12.3	1.1	1.0	8.0	0.6	0.3
Middle Atlantic	**26.9**	72.4	13.9	84.9	56.3	1.6	2.4	19.0	3.7	1.0
South	**22.4**	13.3	35.7	2.2	15.7	3.7	88.9	14.7	4.1	1.3
Midwest	**11.0**	2.1	0.8	0.2	4.7	78.9	2.5	9.9	0.8	1.3
West	**25.1**	1.2	1.2	1.4	6.3	7.8	1.5	40.4	81.6	93.6
International	**6.1**	10.5	6.2	9.5	4.7	6.9	0.4	8.0	9.2	2.5
Missing	**0.5**	0.1	0.0	0.0	0.0	0.0	3.3	0.0	0.0	0.0
**Camp characteristic**										
Persons per cabin, range	**NA**	14–20	8–15	16–17	2–3	15–17	14–20	10–15	14–40	12–16
Length of session, wks (no. of sessions)	**NA**	6.5 (1)	8 (1); 4 (2)^¶^	4 (1); 3 (1)^¶^	2 (3)^¶^	8 (1); 4 (2)^¶^	8 (1); 4 (2)^¶^	4 (2)^¶^	6.5 (1); 2 (3)^¶^	3 (2)^¶^
Local SARS-CoV-2 community transmission**										
At start of camp	**NA**	1.4	0.8	0.9	1.8	1.9	2.5	7.3	2.1	1.1
At intersession	**NA**	NA	6.0	6.3	2.4 (1st); 5.3 (2nd)	NA	4.2	7.3	0 (1st); 4.0 (2nd)	4.4

**Abbreviations:** NA = not applicable; NR = not reported.

* Age at start of camp.

^†^ Vaccinated is defined as ≥2 weeks after completion of the primary vaccination series (i.e., 2 doses of one of the mRNA COVID-19 vaccines [Pfizer-BioNTech or Moderna] or single dose of the Janssen [Johnson & Johnson] COVID-19 vaccine). Documentation (upload of vaccination card) or parent attestation of vaccination was submitted to each camp by the start of camp. Vaccination rates (denominators) only include eight camps that provided vaccination data: camps A, B, C, D, E, G, H, and I.

^§^ Domestic home regions defined according to U.S. Census regions. International included Argentina, Canada, Colombia, Dominican Republic, Great Britain, Hungary, Israel, Jamaica, Japan, Mexico, Netherlands, Poland, United Kingdom, and U.S. military overseas bases. https://www2.census.gov/geo/pdfs/maps-data/maps/reference/us_regdiv.pdf

^¶^ Campers and staff members may stay for multiple sessions.

** Daily new cases per 100,000 population. https://www.covidactnow.org

All camps requested that staff members and campers adhere to masking and physical distancing when interacting with persons outside their immediate family for 10–14 days before arrival at camp. Masking and physical distancing requirements were strongly recommended while traveling to camp. Campers^§^ across all nine camps were required to submit at least one negative SARS-CoV-2 RT-PCR test result from a test performed within 72 hours before the start of camp, regardless of vaccination status.

The frequency and type of screening testing during camp varied across the camps and by vaccination status. In addition to a prearrival RT-PCR screening test, at least three screening tests were required by all camps for unvaccinated campers through the first 12 days after arrival. Six camps used a combination of rapid antigen and RT-PCR testing for screening; the remaining three used only RT-PCR testing for screening. RT-PCR test results were returned within approximately 12–24 hours. One camp performed wastewater RT-PCR surveillance testing three times per week in addition to screening testing.

Frequent testing was coupled with multiple prevention strategies, including podding, masking, physical distancing, and hand washing. A pod began as a group of campers and staff members who were in the same cabin. Pod residents were allowed to interact with each other without masking or physically distancing. Camps merged pods in stages, growing from one cabin to multiple cabins, to age groups. Each session required new campers and staff members to follow the same podding protocol. Three camps reached campwide pod expansion. The decision to end indoor masking at each pod expansion stage was predicated on all persons having a negative test result, unless state or local regulations prevented this (one camp). Staff members were permitted to remove masks when they were among other vaccinated staff members and separated from unvaccinated persons. To facilitate physical distancing, camps maximized outdoor activities, staggered mealtimes, divided persons into groups to eat indoors and outdoors, staggered medication administration,^¶^ segregated infirmary care, and designated sick call by pod.** Hand sanitizing or washing before and after all activities and meals was required and facilitated by increased availability of dispensers and wash stations across camp. Camps varied with regard to whether staff members or campers were permitted off site; three camps permitted staff members to have days off outside of camp and four camps permitted off-site activities for staff members during intersession; only one camp permitted campers off site on one supervised trip. Off-site excursions were supervised in controlled settings or required staff member self-attestation to protocol compliance while off site. Compliance was monitored in-person by senior administrative staff members. If off-site excursions occurred between sessions, screening testing protocols were implemented.

During June–August 2021, a total of 38,059 rapid antigen and RT-PCR prearrival and camp screening tests were performed across the nine camps (Table 2). Screening testing identified 21 persons with positive test results; among these, 15 persons had a positive rapid antigen screening test result who were found to have negative RT-PCR test results. Thus, a total of six persons had SARS-CoV-2 infections confirmed by RT-PCR screening testing, for a screening test positivity rate of 0.02%. Three additional cases were confirmed among symptomatic persons by RT-PCR, yielding a total of nine cases (0.1%) identified across the camp population during the 2021 season. The nine cases occurred at four camps. Three of the nine cases occurred in vaccinated staff members and six in unvaccinated campers aged 8–14 years. The three staff member cases were identified before the arrival of campers. One case in a vaccinated symptomatic staff member occurred during initial staff week, and the other two cases in vaccinated staff members (one asymptomatic, one symptomatic) occurred between sessions. These two cases, which resulted from exposures between sessions and before camper arrival, were attributed to activity outside of camp and occurred when surrounding community case counts had risen two- to sevenfold since the start of camp for six of the seven camps with intersessions (Table 1). Thereafter, off-site activities were cancelled.

**TABLE 2:** SARS-CoV-2 screening testing* — nine U.S. overnight camps, June–August 2021

Timing of testing	No. of rapid antigen and RT-PCR screening tests	No. of persons with RT-PCR–positive screening test results	No. of persons with rapid antigen–positive, RT-PCR–negative screening test results
**Total**	**38,059**	**6**	**15**
**Staff member week**			
Prearrival day –14 or –10	502	—^†^	—
Prearrival day –3	1,547	—	—
Arrival day 0	664	—	—
Day 3	184	—	—
Day 5 or 6	343	—	—
**Session 1**			
Prearrival day –14 or –10	2,407	—	—
Prearrival day –3	4,001	1^§,¶^	—
Day 0	3,749	—	1^¶^
Day 2 or 3	1,675	—	3^§,¶^
Day 4 or 5	2,082	1^§,¶^	1^§,¶^
Day 7 or 8	2,060	1^§,¶^	—
Day 11–16	1,293	—	—
Day 18–22	1,776	—	—
**Intersession**			
Day 0	235	1^¶^	—
Day 3	238	—	—
Day 5	188	—	—
**Session 2**			
Prearrival day –14 or –10	636	—	—
Prearrival day –3	1,592	—	—
Day 0	2,635	1^§,¶^	—
Day 2 or 3	934	—	—
Day 4 or 5	2,150	1^§,¶^	1^¶^
Day 7 or 8	2,418	—	—
Day 11–16	1,796	—	3^¶^, 5^§,¶^
Day 18–22	1,262	—	—
Day 23 or 26	878	—	—
**Session 3**			
Prearrival day –3	151	—	—
Day 0	459	—	1^¶^
Day 3	110	—	—
Day 5	94	—	—

**Abbreviation:** RT-PCR = reverse transcription–polymerase chain reaction.

*Results include all preplanned screening testing according to each camp’s COVID-19 prevention protocol from prearrival staff member week and through camp. Symptomatic and exposure testing results (positive and negative results) are not included. All screening tests had Food and Drug Administration Emergency Use Authorization.

^†^ No confirmed cases or persons with positive rapid antigen and negative RT-PCR test result.

^§^ Unvaccinated (defined as not having received any dose of the COVID-19 vaccine).

^¶^ Asymptomatic.

Two of the six campers with cases were asymptomatic and identified by prearrival screening; these campers did not enter camp. Three additional cases were identified by screening testing, and one was identified because the camper was symptomatic; all were identified within the first 8 days of camp. One camper case was traced to an exposure during an activity outside of camp between sessions. Campers from camps with confirmed cases were either sent home or isolated according to local health department guidance. Camps tested all potentially exposed contacts and varied according to whether all or only unvaccinated contacts were quarantined. No cases were identified at the camp that conducted wastewater surveillance. No secondary transmission was detected during camp. 

## Discussion

Implementation of multicomponent prevention strategies, including achievement of high vaccination coverage among those eligible for vaccination, prearrival and frequent screening testing, and use of additional concomitant prevention measures were critical to limiting the introduction and spread of COVID-19 in overnight youth camps. Frequent screening and testing of symptomatic campers and staff members resulted in rapid identification and isolation of persons with COVID-19 and quarantine of exposed contacts according to local health agency recommendations. Podding aided in containment of potential cases and provided campers the ability to continue to interact with their peers. These multipronged strategies ultimately resulted in no identified cases of secondary transmission during camp. Achieving a successful summer of preventing spread of COVID-19 at these overnight camps required extensive preparation and coordination. The organization’s national medical committee was essential to providing guidance on the myriad prevention strategies and sharing critical real-time experiences throughout the summer.

Several camps permitted staff members to leave camp under specific protocol guidance. These outings increased the risk for SARS-CoV-2 exposure, infection, and transmission. Three of the nine cases resulted from this type of activity, underscoring the importance of vigilance when permitting activity outside the established controlled camp environment. These cases, combined with the rise in transmission across surrounding camp communities, led to the mid-summer cancellation of off-site activities.

The findings in this study are subject to at least two limitations. First, although symptomatic testing was performed according to protocol, and all positive test results were documented, negative results of tests conducted for symptoms or exposure were not always documented because of infirmary staffing challenges. This resulted in an unknown number of total tests performed after arrival. Consequently, results from symptomatic and exposure testing were not included in the test positivity rate, resulting in an overestimation. Second, one camp did not collect documentation of vaccination status among campers. All persons from this camp were removed from vaccination rate results, yet all staff members from this camp attested to full vaccination before the start of camp. As such, the overall vaccination rates and that among persons aged ≥17 years are likely underestimates.

These findings underscore the importance of simultaneous implementation of multicomponent strategies to reduce and prevent the transmission of SARS-CoV-2 in overnight youth camp settings. The combination of high vaccination rates among persons eligible for vaccination, frequent testing, podding, modified programming, masking, physical distancing, and attention to hand hygiene afforded campers and staff members safe engagement with their peers and camp community. These findings also highlight important guiding factors for development and implementation of COVID-19 prevention protocols in other youth-focused settings, including schools and related youth programs.

**Summary**

**What is already known about this topic?**

Previous studies have demonstrated the importance of prevention strategies to reduce SARS-CoV-2 transmission in overnight camps.

**What is added by this report?**

During June–August 2021, a total of 7,173 campers and staff members attended nine U.S. overnight camps that implemented multiple prevention strategies including high vaccination coverage (>93% among eligible persons aged ≥12 years); prearrival and frequent screening testing (38,059 tests); and additional concomitant prevention measures. Nine laboratory-confirmed COVID-19 cases and no secondary infections were detected.

**What are the implications for public health practice?**

Implementation of high vaccination coverage coupled with multiple prevention strategies is critical to averting COVID-19 outbreaks in congregate settings, including overnight camps. These findings highlight important guiding principles for school and youth-based COVID-19 prevention protocols.

## Footnotes

*
https://www.medrxiv.org/content/10.1101/2021.02.18.21250271v1

† Vaccination rates reflect data submitted from eight of nine camps; 4,289 persons aged ≥12 years were eligible for vaccination.

§ Eight camps required staff members to submit a negative SARS-CoV-2 RT-PCR test result upon arrival for staff member week. In the one camp without this requirement, all staff members received rapid antigen testing before arrival of campers.

¶ All medications (prescription or over-the-counter) needed by campers during the summer were required to be administered by medical staff members at the infirmary. Campers were not permitted to keep any medication in their cabin.

** Infirmary sick call followed an established schedule such that specific hours were designated for each pod or group of pods. Exceptions were made for emergency medical circumstances.
